# Description of microscopic lesions of vestibular folds of autopsied adults and their relationship with cause of death and underlying disease

**DOI:** 10.1016/S1808-8694(15)31305-7

**Published:** 2015-10-20

**Authors:** Renata C. Rossi, Ana. K.M. Salge, Rosana R.M. Correa, Mara L.F. Ferraz, Vicente P.A. Teixeira, Marlene A. Reis, Eumenia C.C. Castro

**Affiliations:** ^1^ Physical Therapist, Master studies under course, Post-graduation in General Pathology, Medical School, Triângulo Mineiro; ^2^ Registered Nurse, Ph.D. studies under course, Post-Graduation in General Pathology, Medical School, Triângulo Mineiro; ^3^ Registered Nurse, Master studies under course, Post-Graduation in General Pathology, Medical School, Triângulo Mineiro; ^4^ Biologist, Discipline of General Pathology, Medical School, Triângulo Mineiro; ^5^ Physician, Ph.D. studies under course, Faculty Professor, Discipline of General Pathology, Medical School, Triângulo Mineiro; ^6^ Physician, Ph.D. studies under course, Joint Professor, Discipline of General Pathology, Medical School, Triângulo Mineiro; ^7^ Physician, Post-doctorate studies under course, Joint Professor, Discipline of General Pathology, Medical School, Triângulo Mineir

**Keywords:** adults, autopsy, inflammation, vestibular folds

## Abstract

The increase in invasive methods currently applied to diagnosis airway upper tract infection leads to a possible increase in vestibular folds (VF) lesions. Besides, VF importance in the prevention of the organism against infection pathogens had been stressed and few studies had addressed the microscopic lesions of the VF in autopsied patients because there is no routine VF examination in the postmortem exam.

**Aim:**

The aim of this study is morphological microscopic analyses of the VF from autopsied patients and its correlation with basic disease and cause of death.

**Study design:**

transversal cohort.

**Material and Method:**

We studied 82 larynges collected during the autopsy exam and performed the Hematoxylin -eosin method for morphological analyses.

**Results:**

From the 82 vestibular folds analyzed we observe that 42 (51%) showed an inflammatory reaction. In fifteen (18.3%) vestibular folds we found lymphoid follicular hyperplasia, in eleven (13.4%) diffuse inflammatory infiltrate and in sixteen (19.5%) acute inflammatory reactions. Circulatory diseases were the most frequently underlying diseases found, 31 (37.8%) and from these 20 (67.8%) presented associated vestibular folds inflammatory reaction. The infection diseases were the most frequently cause of death among the patients with inflammatory reaction of the VF.

**Conclusion:**

Besides the anatomic function, VF seem to have a immunological function preventing lower airway infections. Our study demonstrated inflammatory PV reactions in patients with infections diseases as cause of death; this finding could be a consequence of the sepses that leads the patient to death or a different way used by the organism to prevent infection.

## INTRODUCTION

Vestibular folds (VF) are two thick laminae, saggitally positioned with doubled-sized mucosa that emerge from the supraglottic wall and form medially the wall of laryngeal ventricle. The mucosa is recovered by non-stratified squamous epithelium and submucous glands. Squamous epithelium may duplicate and increase withaging^1^, ^2^, ^3^.

Vocal folds are located at the glottis and they are the first barrier to the passage of infectious agents to lower airways. As a result of the increase in invasive methods used for the diagnosis of respiratory tract diseases, it is expectable that VF abnormalities become more frequent. Moreover, the importance of VF in protecting the body against infectious agents has been recently discussed^1^, ^2^. In studies conducted with true vocal folds we can find the description of lesions such as thickness of basal membrane of true vocal folds epithelium and their relation with infectious diseases^4^, but there is no report on vestibular folds. As to microscopic lesions of VF, there are reports of lymphoid follicle hyperplasia of VF and the suggestion of the term laryngeal tonsil^5^ to name the structure, since it has been observed that this tissue presents an organization and that it could be replaced by larynx associated lymphoid tissue^2^. However, all studies were conducted with few patients and no other associated lesions were described.

The studies conducted with VF are scarce and insufficient to make us understand the microscopic lesions found in these organs. The objective of the present study was to describe the microscopic affections to vestibular folds and to associate them with cause of death and underlying disease of autopsied adults.

## MATERIAL AND METHODS

**Figure 1:**
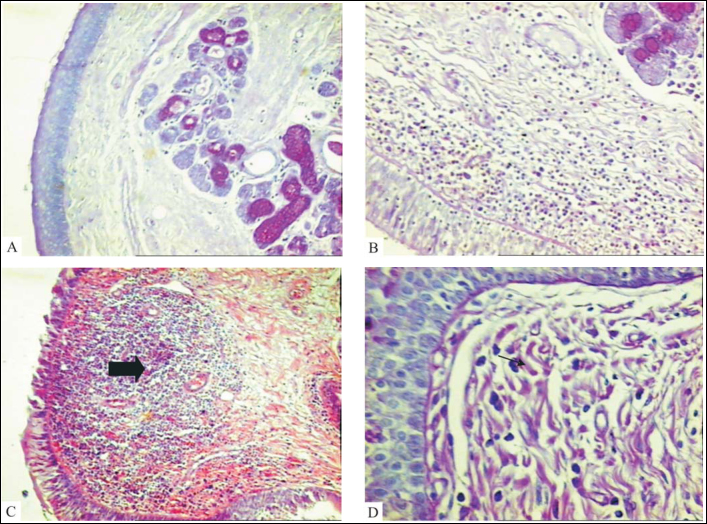
Microscopic characterization of vestibular fold lesions. Normal vestibular fold (A) with discreet diffuse mononuclear infiltrate or absence of inflammatory cells on lamina propria. Vestibular folds with intense mononuclear inflammatory infiltrate on lamina propria (B) and follicle hyperplasia (C, arrow). The presence of neutrophils in the inflammatory infiltrate (D, arrow) was considered as acute inflammatory reaction (PAS, 320X).

We conducted a retrospective transversal study of consecutive autopsies in adults carried out by the Discipline of General Pathology, University Hospital, Medical School, Triângulo Mineiro (FMTM), in Uberaba-MG, between 1993 and 2001. Larynges were removed and all autopsies were fixed with formaldehyde at 10%. Later, the larynx was sectioned transversally at points above and below the glottic cavity and within 3cm one from the other. Parallel sections to the extremity of the vocal folds were made, transversally, and the fragment was processed for paraffin preparation^4^. We performed hematoxylin-eosin staining (HE) for morphological analysis. We considered normal VF (Figure 1A) when they had with discreet diffuse mononuclear cell infiltrate or absence of inflammatory cells on lamina propria. VF that had lymphoid follicle hyperplasia or moderate or severe chronic or acute inflammatory reactions were considered as having inflammatory reaction.

We collected gender and age of patients from medical charts. From autopsy reports, we collected diagnosis of death and underlying diseases. The diseases were grouped according to the International Classification of Diseases^6^. The present study was approved by the Research Ethics Committee, Medical School, Triângulo Mineiro.

To conduct statistical analysis, we used software Sigmastat. Proportions were compared with χ^2^, plus Fisher exact test. For the correlation between variable of normal distribution, we used Pearson correlation coefficient, if not, we applied Spearman coefficient. Differences were considered statistically significant when *p* was smaller than 5% (*p* < 0.05).

## RESULTS

We analyzed 82 VF from autopsied patients. Mean age of patients was 53.0 ± 16.7 years and 48 (59%) were male. There were 40 normal VF (48.8%) and 42 cases (51%) presented morphological abnormalities.

The morphological affection found in the VF was inflammatory reaction of variable severity. We found diffuse marked mononuclear inflammatory infiltrate on the lamina propria of VF in 11 patients (13.4%) (Figure 1B). Hyperplasia of lymphoid follicles was found in 15 cases (18.5%) (Figure 1C). The presence of neutrophils in the inflammatory infiltrate was considered as an acute inflammatory reaction (Figure 1D) and it was diagnosed in 16 (19.5%) of the patients (Table 1). We did not find any other microscopic affections of VF.

**Table 1:** Description of microscopic findings of vestibular folds, age and gender of autopsied patients at University Hospital, Medical School, Triângulo Mineiro, in Uberaba-MG, between 1993 and 2001.

Groups	n (%)	*X–SD (years)	Gender n (%)	
			Male	Fem
Normal	40 (48.8)	53.7–18.9	23(57.5)	17(42.5)
Acute inflammatory reaction	16 (19.5)	56.4–13.5	11(69)	5(31)
Lymphoid follicle hyperplasia	15 (18.3)	50.3–14.4	8(53)	7(47)
Diffuse inflammatory infiltrate	11 (13.4)	49.8–16.3	6(55)	5(45)
Total	82 (100)	53.0–16.7	48(59)	34(41)

^*^ F=0.497; p=0.685

As to the group of underlying diseases, we observed that the group of patients that presented normal VF were classified as having infectious and parasitic diseases - 14 cases (35%), and AIDS was diagnosed in 12 cases, the most frequent disease. To patients that presented inflammatory reaction, 20 (48%) had circulatory system diseases, and 8 of them (40%) had chronic Chagas disease (Table 2).

**Table 2:** Description of microscopic affections to vestibular folds concerning underlying diseases in autopsied patients at University Hospital, FMTM, in Uberaba-MG, between 1993 and 2001

Group Normal			Group Inflammatory Reaction		
	Underlying disease	n (%)	GDB	Underlying disease	n (%)
DAC (n = 12)	CH	4 (10)	DAC (n = 20)	CC	8 (19)
	CC	3 (7.5)		CH	7 (16.6)
	Atherosclerosis	1 (2.5)		Atherosclerosis	1 (2.4)
	CR	1 (2.5)		CVA	1 (2.4)
	Cor Pulmonale	1 (2.5)		CR	1 (2.4)
	C. Pulmonale Chronic	1 (2.5)		COPD	1 (2.4)
	COPD	1 (2.5)		ARDS	1 (2.4)
DIP (n = 14)	AIDS	12 (30)	DIP (n = 10)	AIDS	9 (21.4)
	Suppurated Appendicitis	1 (2.5)		Purulent Peritonitis	1 (2.4)
	Paracoccidioidomycosis	1 (2.5)			
NEO (n = 5)	Lymphoma	2 (5.0)	NEO (n = 8)	Hepatocarcinoma	2 (4.8)
	Esophageal Carcinoma	1 (2.5)		LMA	2 (4.8)
	Rectum Carcinoma	1 (2.5)		Gastric Adenoma	1 (2.4)
	Colangiocarcinoma	1 (2.5)		AG	2 (4.8)
				AC	1 (2.4)
TMC (n = 4)	Alcoholic Cirrhosis	3 (7.5)	TMC (n = 2)	Chronic alcohol abuse	2 (4.8)
	Chronic alcohol abuse	1 (2.5)			
DAD (n = 2)	AAO	1 (2.5)	DAD (n = 2)	SIO	1 (2.4)
	Perforated ulcer	1 (2.5)		PAN	1 (2.4)
Others		3 (7.5)			
Total		40 (100)			42 (100)

AAO: obstructive acute abdomen, AG: gastric Adenocarcinoma, AC: colon adenocarcinoma, CVA: cerebral vascular accident, CC: Chagas Cardiopathy, CH: Hypertensive Cardiopathy: CR: Rheumatic Cardiopathy; DAC: circulation system disease, DAD: digestive system diseases, DIP: infectious and parasitic diseases, COPD: chronic obstructive pulmonary disease, LMA: acute myeloid leukemia, NEO: neoplasms, TMC: mental and behavioral disorders, PAN: necrotising acute pancreatitis. ARDS: acute respiratory distress syndrome, SIO: obstructive icteric syndrome.

As to cause of death, we detected that infectious and parasitic diseases were the most frequent cause of death both for the normal group (21 - 52%) and the inflammatory reaction group (20 - 50%), being that pneumonitis was the most frequent disease (Table 3).

**Table 3:** Description of microscopic affections of vestibular folds concerning cause of death diagnosed in autopsied patients at Hospital, FMTM, in Uberaba-MG, between 1993 and 2001

Group Normal			Group Inflammatory Reaction		
GCM	Cause of death	n (%)	GCM	Cause of death	n (%)
DIP (n = 21)	Bronchopneumonia	13 (32.5)	DIP (n = 21)	Bronchopneumonia	14 (33.3)
	Purulent Peritonitis	4 (10)		Epicarditis	2 (4.8)
	ICG	3 (7.5)		EIB	1 (2.4)
	EIB	1 (2.5)		ICG	2 (4.8)
				Purulent Peritonitis	1 (2.4)
				TC	1 (2.4)
DAC (n = 10)	TP	2 (5.0)	DAC (n = 8)	Pulmonary Edema	4 (9.5)
	CVA	1 (2.5)		CI	1 (2.4)
	Cor Pulmonale	1 (2.5)			
	Cerebral Edema	1 (2.5)		Pulmonary Infarction	1 (2.4)
	Pulmonary Edema	1 (2.5)		Pulmonary emphysema	1 (2.4)
	Pulmonary Embolism	1 (2.5)		TP	1 (2.4)
	EHP	1 (2.5)			
	ICC	1 (2.5)			
	PCAD	1 (2.5)			
DAD (n = 3)	PAN	2 (5.0)	DAD (n = 9)	EHI	2 (4.8)
	HD	1 (2.5)		Alcohol Hepatitis	2 (4.8)
				PAN	2 (4.8)
				EAUP	1 (2.4)
				Gastroenterorrhage	1 (2.4)
				MA	1 (2.4)
NEO (n = 3)	LNH	1 (2.5)	NEO (n = 2)	AP	1 (2.4)
	Metastases	1 (2.5)		LMA	1 (2.4)
Others (n = 3)		3 (7.5)	Other (n = 2)		2 (2.4)
Total		40 (100)			42 (100)

AP: pulmonary adenocarcinoma, CH: Hypertensive Cardiopathy, CI: ischemic cardiopathy, DAC: circulation system disease, DAD: digestive tract diseases, DIP: infectious and parasitic diseases, EAUP: perforated ulcerated acute esophagitis, EHI: intestinal hemorrhagic infarction, EHP: pulmonary hemorrhagic infarction, EIB: bacterial infectious endocarditis, GCM: group cause of death, HD digestive hemorrhage, ICC: congestive heart failure, ICG: chronic granulomatous infections, LMA: acute myeloid leukemia, LNH. Non-Hodgkin lymphoma, MA: marked megacolon, NEO: neoplasm, PAN: necrotising acute pancreatitis, PCAD: diffuse marked chronic pleuritis, TC: cerebral toxoplasmosis, TP: pulmonary thromboembolism.

Intense and diffuse inflammatory reaction in 15 (55.5%) of the cases was found as more frequent among patients that had died of infectious diseases, regardless of underlying disease (table 4).

**Table 4:** Description of groups of Cause of Death relative to microscopic lesions found in vestibular folds of adult patients autopsied at University Hospital, Medical School, Triângulo Mineiro, in Uberaba-MG, between 1993 and 2001.

Group Cause of death	Cases n (%)	NL n (%)	HFL n (%)	RIA and RID n (%)
DIP	42 (51.2)	21 (52.5)	6 (40)	15 (55.5)
DAC	18 (21.9)	10 (25)	4 (26.6)	4 (14.8)
DAD	12 (14.6)	4 (7.5)	5 (33.3)	4 (14.8)
NEO	5 (6.0)	3 (7.5)	0 (0)	2 (7.4)
CEMM	2 (2.4)	1 (2.5)	0 (0)	1 (3.7)
DAGU	2 (2.4)	1 (2.5)	0 (0)	1 (3.7)
DSN	1 (1.2)	1 (2.5)	0 (0)	0 (0)
TOTAL	82 (100)	40 (100)	15 (100)	27 (100)

DIPXHFL: χ^2^ = 0.457; p = 0.499. CEMM: external causes of morbidity and mortality, DAC: circulatory system diseases, DAD: digestive tract diseases, DAGU: genital-urinary tract diseases, DIP: infectious and parasitic diseases, DSN: nervous system diseases, HFL: lymphoid follicle hyperplasia, NL: normal, NEO: neoplasms, RIA: acute inflammatory reaction, RID: diffuse inflammatory reaction.

Out of the total patients who had infectious or parasitic causes of death, 21 (25.6%) cases presented AIDS as underlying disease. Out of these cases, the diagnosis of VF inflammatory reaction was made in 5 cases (6%) and hyperplasia of lymphoid follicles in 4 (4.8%), less frequent than in the other patients (Table 5).

**Table 5:** Comparison between patients with AIDS as underlying disease and the other groups of underlying diseases in relation to lesions found in vestibular folds of patients autopsied at University Hospital, Medical School, Triângulo Mineiro, in Uberaba- MG, between 1993 and 2001.

Underlying diseases	Cases n (%)	NL n (%)	HFL n (%)	RIA and RID n (%)
Others	61 (74.4)	28 (70)	11 (73.3)	22 (81.5)
AIDS	21 (25.6)	12 (30)	4 (26.7)	5 (18.5)
TOTAL	82 (100)	40 (100)	15 (100)	27 (100)

ξ^2^ = 0.404; p= 0.525. HFL: lymphoid follicle hyperplasia, NL: normal, RIA: Acute inflammatory reaction, RID: Diffuse inflammatory reaction.

## DISCUSSION

The morphological lesion found in the VF was inflammatory reaction, followed by lymphoid follicle hyperplasia. According to the literature, this is a common finding in VF, but we did not find the described percentages to compare with our data^2^.

VF function is to lubricate and isolate the true vocal folds to allow vibration^7^. Therefore, given that they are neighboring structures and functionally interrelated, we would expect similar lesions. In a study conducted before, different lesions were described from that of true vocal folds 1, but this fact was different concerning our VF because we only found inflammatory affections. These data are in accordance with the hypothesis that there are immunologically different regions in the larynx and that they respond to stimulus in specific ways^1^, ^2^

Inflammatory and diffuse reactions were more frequently found in patients who had died of infectious diseases, regardless of the underlying disease. There are studies that show production of cytokines in the presence of infectious agents in VF^1^, ^2^. Our findings reinforce the hypothesis that VF may be involved in the defense of the organism in view of infections. Another hypothesis is that the demonstrated inflammatory reaction is part of systemic septic reaction that resulted in death of the patient.^8^

Both the diffuse inflammatory reaction and lymphoid follicle hyperplasia were found as less frequent in immunodepressed subjects. These patients present generalized immunodeficiency, which may justify the lack of inflammatory infiltrate in VF of this group of patients.

## CONCLUSION

In addition to the anatomical function, VF have a possible immune function concerning upper airway infections. Our study showed inflammatory reaction in vocal folds of patients with infectious and parasitic diseases as cause of death, which may be a finding related with generalized septic process that led the subject to death or be one of the forms used by the body to prevent penetration of infectious agents through the lower airways
